# A consistent discretization via the finite radon transform for FFT-based computational micromechanics

**DOI:** 10.1007/s00466-024-02542-9

**Published:** 2024-09-14

**Authors:** Lukas Jabs, Matti Schneider

**Affiliations:** 1 Institute of Engineering Mathematics, University of Duisburg-Essen, Essen, Germany; 2 Fraunhofer Institute for Industrial Mathematics ITWM, Kaiserslautern, Germany

**Keywords:** Computational homogenization, FFT-based computational micromechanics, Discretization, Radon transform, Laminates

## Abstract

This work explores connections between FFT-based computational micromechanics and a homogenization approach based on the finite Radon transform introduced by Derraz and co-workers. We revisit periodic homogenization from a Radon point of view and derive the multidimensional Radon series representation of a periodic function from scratch. We introduce a general discretization framework based on trigonometric polynomials which permits to represent both the classical Moulinec-Suquet discretization and the finite Radon approach by Derraz et al. We use this framework to introduce a novel Radon framework which combines the advantages of both the Moulinec-Suquet discretization and the Radon approach, i.e., we construct a discretization which is both convergent under grid refinement and is able to represent certain non-axis aligned laminates exactly. We present our findings in the context of small-strain mechanics, extending the work of Derraz et al. that was restricted to conductivity and report on a number of interesting numerical examples.

## Introduction

### State of the art

Computational micromechanics is used to accelerate the development of tailored materials for industrial processes, side-stepping the typically resource-intensive manufacturing and prototyping process to a large degree. The key player in this regard is the microstructure, which encodes the pattern in which the individual materials constituting the composite are assembled. However, microstructures of industrial interest typically involve a serious degree of complexity as a result of their manufacturing process.

To handle the complexity of the involved microstructures, avoiding the rather involved meshing procedure for creating interface-conforming finite-element meshes, Moulinec-Suquet [1, 2] introduced a computational approach to periodic homogenization problems in mechanics based on the fast Fourier transform (FFT). Their strategy was able to handle nonlinear and inelastic constitutive behavior from the start and was continuously improved by various authors.

In a nutshell, the success of Moulinec-Suquet’s approach hinges on three factors. For a start, the scheme involves an ingenious fuse of discretization scheme and solution method. Secondly, the computational method is matrix-free and thus permits to solve large-scale computational homogenization problems on moderate hardware, avoiding the necessity to use high-performance computing hardware (which tends to come with its own challenges). Last but not least, the fast Fourier transform is a well-studied and highly optimized Algorithm [3]. In particular, FFT-based computational homogenization methods are *fast*, as their runtime scales as $n\,\log n$ which is almost optimal in term of the total number of voxels *n*.

As already indicated, a rather large ecosystem of derived solution and discretization schemes has been developed in the last decades [4–6], permitting to solve large scale, high or even infinite contrast and nonlinear/inelastic problems with confidence. For example, through the integration of alternative discretizations into the framework it was possible to mitigate certain shortcomings of the original approach: Discretizations based on finite differences [7–9] or finite elements [10–12] lead to fewer artifacts than the Moulinec-Suquet discretization and ensure convergence of Lippmann-Schwinger solvers for stable porous materials [13]. Fourier-Galerkin discretizations [14–16], on the other hand, permit to compute upper and lower bounds of the effective tensors arising for linear composites.

Additionally, more powerful solvers were incorporated with the objective of enhancing the convergence behavior and robustness in comparison to the original basic scheme [1, 2], e.g., strategies that employ Newton’s method [17–19] or quasi-Newton schemes [20–22], fast gradient techniques [23–25], Krylov methods [26–28] or polarization schemes which come in two flavors: Eyre-Milton type methods [29–31] and approaches based on ADMM [32–34]. Recent work on FFT-based methods focused on local adaptivity [35–37], mesh-morphing techniques [17, 38, 39] and the consideration of alternative boundary conditions, both for conductivity [40–43] and mechanics [44–46].

Despite all these changes, the fast Fourier transform and its closely related real transforms like cosine and sine transforms has always remained the central part of the method. Only recently, Derraz et al. [47] proposed a different approach to (thermal) computational homogenization problems based on the finite Radon transform [48, 49] which has a number of similarities with, but also a few differences to Moulinec-Suquet’s original approach [1, 2].

### Contributions

This work had its starting point in the first author’s MSc thesis, which intended to connect the work of Derraz et al. [47] to the mainstream of computational micromechanics. The finite Radon transform [48, 49] is a discrete and periodic version of the Radon transform which considers the averages of a given (sufficiently smooth) function over any hyperplane through the origin. Gelfand et al. [50] presented a periodic version of the Radon transform where periodic functions are considered and the average is calculated over suitably wrapped hyperplanes. In fact, Gelfand et al. [50] considered the two-dimensional setting and provided a suitable Radon transform, together with its inverse. A finite version of the Radon transform was introduced by Matúš-Flusser [48] in two dimensions and for an $N \times N$-grid where *N* is a prime number, which was subsequently generalized and extended [51–53]. The focus of these approaches was image analysis, i.e., decomposing a given image into its Radon projections, and using the Radon transform as a convenient means to perform common image-processing tasks, e.g., filtering. A key component of both the continuous and the finite Radon transform is its close connection with the Fourier transform (and its discrete cousin), oftentimes referred to as the Fourier slice theorem.

After recalling the fundamentals of periodic homogenization, the work at hand provides a direct route to the finite Radon series, generalizing the formula given by Gelfand et al. [50] to arbitrary dimensions, see Sect. 2. Our strategy is based on the Fourier slice theorem, and derives the Radon series from combinatorial considerations. In fact, the Fourier version of the Radon series holds in greater generality than its averaging version, which we also derive in a straightforward way.

Subsequently, we turn our attention to the finite Radon transform [48, 49]. We give a self-contained development in Sect. 3.1, valid for any dimensions, but restricted to voxel counts per axis which are prime. In fact, there may be generalizations to other voxel counts in the spirit of the two-dimensional works [51–53]. However, to avoid case distinctions and therefore increase readability we restrict to prime voxel counts per dimension for the work at hand. This restriction is rooted in the correspondence with Cartesian products of fields with prime characteristic where any two distinct points uniquely determine a line - like for real numbers!

We proceed to derive the finite Radon discretization [48, 49] from first principles, with the added benefit that we treat mechanical problems in arbitrary dimensions naturally. Our derivation, detailed in Sect. 3.2, permits us to point out a fundamental problem of the finite Radon discretization [47]. More precisely, the discretizations requires selecting an element which generates the corresponding line (whose level sets determine the hypersurfaces). In contrast to the continuous case, there is a large number of different generators to chose from. In fact, any non-zero point on the line can be chosen as the generator! The finite Radon discretization associates an Eshelby-Green operator to each such line and sets up Moulinec-Suquet’s basic scheme accordingly. However, it turns out that different choices of generators may lead to different Eshelby-Green operators!

With this insight at hand, we are led to the conjecture that the discretization introduced by Derraz et al. [47] may not be convergent under mesh refinement. We confirm this suspicion with a well-selected computational experiment in Sect. 4.3. After identifying the source responsible for the lack of convergence, we propose a consistent version of the Radon discretization in Sect. 3.3.

The work at hand culminates in a few computational experiments, see Sect. 4, which illuminate both the advantages and the limitations of Radon-based discretizations.

## Periodic homogenization via the radon series

### Periodic homogenization

We are concerned with a periodic homogenization problem in linear elasticity, i.e., we consider a rectangular unit cell

2.1 $$\begin{aligned} Y = [0,L_1] \times [0,L_2] \times \cdots \times [0,L_d] \end{aligned}$$

and suppose that a linear elastic stiffness tensor

2.2 $$\begin{aligned} \mathbb {C}: Y \rightarrow L(\text {Sym}(d)) \end{aligned}$$

is given, where $\text {Sym}(d)$ stands for the vector space of symmetric $d \times d$ tensors and *L*(*V*) refers to the set of linear transformations on the vector space *V*. We assume that the stiffness distribution (2.2) is symmetric (almost) everywhere, bounded uniformly and the smallest eigenvalue is bounded away from zero uniformly.

For a given macroscopic strain tensor $\bar{\varvec{\varepsilon }}$, we seek a periodic displacement fluctuation $\varvec{ u}\in H^1_{\text {per}}(Y)^d$, a strain field $\varvec{\varepsilon }\in L^2(Y;\text {Sym}(d))$ and a stress field $\varvec{\sigma }\in L^2(Y;\text {Sym}(d))$, s.t. the kinematic compatibility condition

2.3 $$\begin{aligned} \varvec{\varepsilon }= \bar{\varvec{\varepsilon }} + \nabla ^s \varvec{ u}\end{aligned}$$

involving the symmetrized gradient operator $\nabla ^s$, the linear elastic constitutive law

2.4 $$\begin{aligned} \varvec{\sigma }= \mathbb {C}: \varvec{\varepsilon }\end{aligned}$$

and the equilibrium condition without body forces

2.5 $$\begin{aligned} \text {div }\varvec{\sigma }= \varvec{0} \end{aligned}$$

are satisfied. It is customary to eliminate both the strain and the stress field from these conditions, leading to the compact equation

2.6 $$\begin{aligned} \text {div }\mathbb {C}:\left( \bar{\varvec{\varepsilon }} + \nabla ^s \varvec{ u}\right) = \varvec{0} \end{aligned}$$

for the displacement-fluctuation field $\varvec{ u}$. Under our assumptions on the stiffness distribution $\mathbb {C}$, there exists a solution $\varvec{ u}$ to the problem (2.6), unique up to the addition of a constant vector.

Treating the prescribed strain $\bar{\varvec{\varepsilon }}$ as a parameter, and making this parameter-dependence explicit for the displacement fluctuation $\varvec{ u}_{\bar{\varvec{\varepsilon }}}$, the effective stiffness $\mathbb {C}^{\text {eff}}$ is defined by volume-averaging the microscropic stress field (2.4)

2.7 $$\begin{aligned} \mathbb {C}^{\text {eff}}:\bar{\varvec{\varepsilon }} = \left\langle { \mathbb {C}:(\bar{\varvec{\varepsilon }} + \nabla ^s \varvec{ u}_{\bar{\varvec{\varepsilon }}})} \right\rangle _Y, \end{aligned}$$

where the operator $\left\langle {\cdot } \right\rangle _Y$ computes the volume average of the quantity in brackets.

### Lippmann-Schwinger reformulation

It is customary in micromechanics to reformulate the equilibrium equation (2.6) in terms of a Lippmann-Schwinger equation [54–56]

2.8 $$\begin{aligned} \varvec{\varepsilon }= \bar{\varvec{\varepsilon }} - \Gamma ^0 : (\mathbb {C}- \mathbb {C}^0): \varvec{\varepsilon }\end{aligned}$$

by introducing a homogeneous reference material $\mathbb {C}^0$ and the associated Eshelby-Green operator

2.9 $$\begin{aligned} \Gamma ^0 = \nabla ^s \left( \text {div }\mathbb {C}^0:\nabla ^s \right) ^\dagger \text {div }, \end{aligned}$$

where $(\cdot )^\dagger$ denotes the Moore-Penrose pseudoinverse. In fact, due to the rectangular shape of the cell (2.1) and the imposed periodic boundary conditions on the displacement fluctuation $\varvec{ u}$, working with Fourier series representations of the involved fields turns out to be quite favorable. Recall that a square-integrable field $\phi :Y \rightarrow \mathbb {R}$ may be written in terms of a Fourier series

2.10 $$\begin{aligned} \phi (\varvec{ x})= &   \sum _{\xi \in \mathbb {Z}^d} \hat{\phi }(\varvec{\xi })\,\exp \left( i \, \varvec{ x}\cdot \tilde{\varvec{\xi }}\right) \quad \text {with} \quad \tilde{\varvec{\xi }}_k = \frac{2\pi }{L_k} \xi _k \nonumber \\  &   \quad (k=1,2,\ldots ,d), \end{aligned}$$

where the sum is understood in an $L^2$-sense and the Fourier coefficients $\hat{\phi }$ are defined via

2.11 $$\begin{aligned} \hat{\phi }(\varvec{\xi }) = \left\langle { \phi \exp \left( -i \, \varvec{ x}\cdot \tilde{\varvec{\xi }}\right) } \right\rangle _Y \quad \text {for} \quad \varvec{\xi }\in \mathbb {Z}^d. \end{aligned}$$

With this notation at hand, the action of the Eshelby-Green operator on a polarization stress field $\varvec{\tau }\in L^2(Y;\text {Sym}(d))$ may be represented in the form

2.12 $$\begin{aligned} \Gamma ^0:\varvec{\tau }(\varvec{ x}) = \sum _{\xi \in \mathbb {Z}^d} \hat{\Gamma }^0(\varvec{\xi }):\hat{\varvec{\tau }}(\varvec{\xi })\,\exp \left( i \, \varvec{ x}\cdot \tilde{\varvec{\xi }}\right) \end{aligned}$$

with the tensor-valued Fourier coefficients

2.13 $$\begin{aligned} \hat{\Gamma }^0(\varvec{\xi }):\varvec{ A}= \tilde{\varvec{\xi }}\otimes _S\left[ \hat{\varvec{ G}}^0(\varvec{\xi })(\varvec{ A}\cdot \tilde{\varvec{\xi }})\right] \quad \text {for} \quad \varvec{ A}\in \text {Sym}(d) \end{aligned}$$

in case $\varvec{\xi }\ne \varvec{0}$ and $\hat{\Gamma }^0(\varvec{0}) = \varvec{0}$. In the formula (2.13), the $d \times d$-tensor $\hat{\varvec{ G}}^0(\varvec{\xi })$ is defined via

2.14 $$\begin{aligned} \left[ \hat{\varvec{ G}}^0(\varvec{\xi })\right] ^{-1} \varvec{ v}= \tilde{\varvec{\xi }} \cdot \mathbb {C}^0 : \left[ \tilde{\varvec{\xi }}\otimes _s\varvec{ v}\right] \quad \text {for arbitrary} \quad \varvec{ v}\in \mathbb {R}^d. \end{aligned}$$

The Lippmann-Schwinger equation (2.8) is in fixed-point form and gives rise to the suggestive iterative scheme [1, 2]

2.15 $$\begin{aligned} \varvec{\varepsilon }^{k+1} = \bar{\varvec{\varepsilon }} - \Gamma ^0 : (\mathbb {C}- \mathbb {C}^0): \varvec{\varepsilon }^k, \quad k=0,1,\ldots , \end{aligned}$$

for any initial value $\varvec{\varepsilon }^0 \in L^2(Y;\text {Sym}(d))$. The convergence properties of the basic scheme (2.15) depend strongly on the chosen reference material $\mathbb {C}^0$, and we refer to the relevant literature [4–6] for a discussion on selecting this numerical parameter in a salient way.

### The Radon series

Less well-known than the Fourier-series representation is the following fact. There is a set $\mathcal {G}^d\subseteq \mathbb {Z}^d$ of generators, s.t. for every (periodic) square-integrable function $f\in L^2(Y)$ on the cell *Y*, there are one-dimensional (periodic) square-integrable functions

2.16 $$\begin{aligned} \check{f}_{\varvec{\xi }}\in L^2(0,2\pi ) \quad \text {for all} \quad \varvec{\xi }\in \mathcal {G}^d, \end{aligned}$$

s.t. the representation

2.17 $$\begin{aligned} f(\varvec{ x}) = \left\langle {f} \right\rangle _Y + \sum _{\varvec{\xi } \in \mathcal {G}^d } \left( \check{f}_{\varvec{\xi }}\left( \varvec{ x}\cdot \tilde{\varvec{\xi }}\right) - \left\langle {f} \right\rangle _Y \right) \quad \text {for} \quad \varvec{ x}\in Y, \end{aligned}$$

holds in an $L^2$-sense. We call the formula (2.17) the Radon series of the function *f*.

**Fig. 1:**
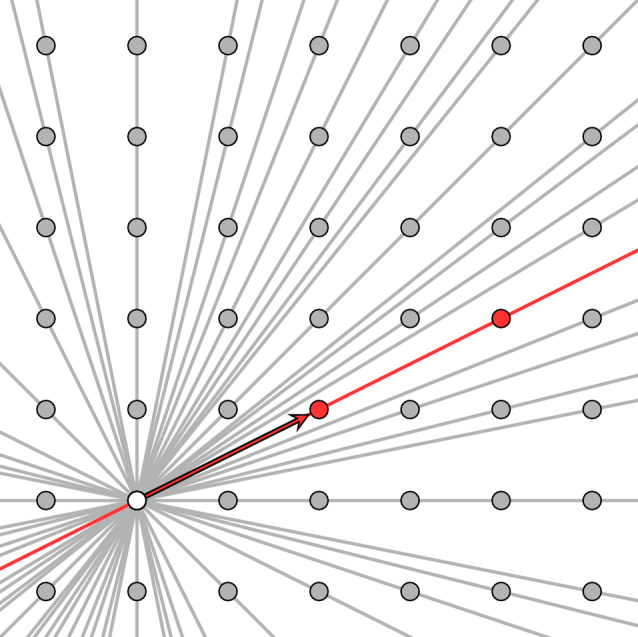
Illustrating the set of Fourier frequencies $\mathbb {Z}^2$ (gray dots) in two spatial dimensions and its decomposition into lines. One line is highlighted in red, corresponding to the generator $\varvec{\xi }=(2,1)$

Before continuing, we collect a few remarks.

1. The potential generators $\varvec{\xi }\in \mathcal {G}^d$ are comprised by all *d*-tuples of integers $\varvec{\xi }\in \mathbb {Z}^d \backslash \{ \varvec{0}\}$ whose non-zero components are coprime. In fact, there are two generators $\varvec{\xi }$ and $-\varvec{\xi }$ which are equivalent, i.e., give rise to essentially the same function (2.16). We select one of these generators by enforcing the first non-zero component of the vector $\varvec{\xi }$ to be positive.
  The sets generated by the generators are illustrated for $d=2$ dimensions in Fig. 1.
2. There are different ways to represent the functions (2.16). For the general case (2.17), i.e., in case the function *f* is square-integrable, we may express the functions (2.16) explicitly
  2.18 $$\begin{aligned} \check{f}_{\varvec{\xi }}(s) = \sum _{k \in \mathbb {Z}} \hat{f}(k \, \varvec{\xi }) \, e^{i ks}, \quad s \in [0,2\pi ], \end{aligned}$$
  in terms of the Fourier coefficients $\hat{f}$ of the original function $f\in L^2(Y)$, compare Eq. (2.11). The formula (2.18) is oftentimes called Fourier slice theorem [50, §1.5].
3. In case the function *f* is sufficiently regular, i.e., $f \in W^{1,1}(Y)$, one may alternatively express the functions $\check{f}_{\varvec{\xi }}$ in the form 2.19 of a $(d-1)$-dimensional average over the *Y*-periodic hyperplane
  2.20 $$\begin{aligned}  &   H_{\varvec{\xi },s} = \left\{ \varvec{ x}\in Y \,\bigg |\, \varvec{ x}\cdot \tilde{\varvec{\xi }} = s\,\,  \texttt { mod }\,\, 2\pi \right\} \quad \text {for} \quad \varvec{\xi }\in \mathcal {G}^d \nonumber \\  &   \quad \text {and} \quad s\in [0,2\pi ) \end{aligned}$$
  involving the modulo operator $\texttt {mod}$. The formula (2.19) justifies the name Radon series for the expression (2.16), as it may be considered as a discrete version of the Radon transform [50, Ch. 1] which involves averages of functions over hyperplanes. Interestingly, the Fourier slice formula (2.18) turns out to be more general than the Radon representation (2.20), as restricting a general $L^2$-function *f* to a hyperplane is not well-defined.
4. The functions (2.16) were presumably first considered by Gelfand et al. [50, §5.3] in the two-dimensional setting and subsequently generalized [49]. We give a brief self-contained derivation of the series representation (2.17) in Appendix A.
5. The Radon series representation (2.17) has applications to generating periodic bicontinuous microstructures [57–59].
6. As Fourier series encode certain higher differentiability properties of functions, the Radon series (2.17) may be used to encode such smoothness properties, as well. For instance, we have the equivalence
  2.21 $$\begin{aligned} f \in H^1_{\text {per}}(Y) \quad \iff \quad \hat{f}_{\varvec{\xi }} \in H^1_{\text {per}}(0,2\pi ) \quad \text {for all} \quad \varvec{\xi }\in \mathcal {G}^d. \end{aligned}$$
  Moreover, for such a Sobolev function, the formula
  2.22 $$\begin{aligned} \nabla f(\varvec{ x}) = \sum _{\varvec{\xi } \in \mathcal {G}^d } \tilde{\varvec{\xi }}\,\check{f}'_{\varvec{\xi }}\left( \varvec{ x}\cdot \tilde{\varvec{\xi }}\right) \end{aligned}$$
  for the gradient of the function *f* at $\varvec{ x}\in Y$ is immediate.

The Radon series (2.17) is very convenient for periodic homogenization problems (2.6). In fact, representing the displacement-fluctuation field $\varvec{ u}$ and the strain field $\varvec{\varepsilon }$ as Radon series, we may recast the compatibility condition (2.3) in the form

2.23 $$\begin{aligned} \varvec{\varepsilon }(x) = \bar{\varvec{\varepsilon }} + \sum _{\varvec{\xi } \in \mathcal {G}^d} \tilde{\varvec{\xi }} \otimes _s \check{\varvec{ u}}'_{\varvec{\xi }} \left( \varvec{ x}\cdot \tilde{\varvec{\xi }} \right) . \end{aligned}$$

Similarly, the balance law for the Cauchy stress (2.5) attains the form

2.24 $$\begin{aligned} \tilde{\varvec{\xi }} \cdot \check{\varvec{\sigma }}_{\varvec{\xi }}'\left( \varvec{ x}\cdot \tilde{\varvec{\xi }} \right) = \varvec{0} \quad \text {for all} \quad \varvec{\xi }\in \mathbb {Z}^d \backslash \{\varvec{0}\}. \end{aligned}$$

Last but not least, as the Fourier-coefficients of the Eshelby-Green operator (2.9) are zero-homogeneous, i.e., satisfy the condition

2.25 $$\begin{aligned} \hat{\Gamma }^0(k\,\varvec{\xi }) = \hat{\Gamma }^0(\varvec{\xi }) \quad \text {for all} \quad \varvec{\xi }\in \mathbb {Z}^d \quad \text {and} \quad k \in \mathbb {Z}\backslash \{\varvec{0}\}, \end{aligned}$$

the Lippmann-Schwinger equation (2.8) (and, in turn, the basic scheme (2.15)) may be treated using the Radon series (2.17), bypassing the complex route inherent to the traditional treatment [1, 2] required by using Fourier series (2.13).

To be more precise, one may progress as follows:

2.26 $$\begin{aligned} \varvec{\tau }^k&= \left( \mathbb {C}- \mathbb {C}^0\right) :\varvec{\varepsilon }^k, \phantom {\sum _{\ell \in \mathbb {Z}}} \end{aligned}$$

2.27 $$\begin{aligned} \check{\varvec{\tau }}^k_{\varvec{\xi }}(s)&= \sum _{\ell \in \mathbb {Z}} \hat{\varvec{\tau }}^k(\ell \, \varvec{\xi }) \, e^{i \ell s} \quad \text {for} \quad s \in [0,2\pi ) \quad \text {and} \quad \varvec{\xi }\in \mathcal {G}^d, \phantom {\sum _{\ell \in \mathbb {Z}}} \end{aligned}$$

2.28 $$\begin{aligned} \check{\varvec{\varepsilon }}^{k+1}_{\varvec{\xi }}(s)&= \bar{\varvec{\varepsilon }} + \Gamma ^0(\varvec{\xi }):\check{\varvec{\tau }}^k_{\varvec{\xi }}(s) \, \text {for} \, s \in [0,2\pi ) \, \text {and} \, \varvec{\xi }\in \mathcal {G}^d, \phantom {\sum _{\ell \in \mathbb {Z}}} \end{aligned}$$

2.29 $$\begin{aligned} \varvec{\varepsilon }^{k+1}(\varvec{ x})&= \bar{\varvec{\varepsilon }} + \sum _{\varvec{\xi } \in \mathcal {G}^d } \left( \check{\varvec{\varepsilon }}^{k+1}_{\varvec{\xi }}\left( \varvec{ x}\cdot \tilde{\varvec{\xi }}\right) - \bar{\varvec{\varepsilon }}\right) \quad \text {for} \quad \varvec{ x}\in Y, \end{aligned}$$

i.e., one first computes the stress polarization in real space, then identifies the Radon projections of the stress polarization, evaluates the action of the Eshelby-Green operator on the Radon projections and finally uses the Radon series (2.17) to synthesize the next iterate. If the formula (2.20) applied, we would thus arrive at a purely real Lippmann-Schwinger iterative solver. Unfortunately, however, the regularity of the involved fields is too low, and it is required to resort to Fourier series (2.10) (such a restriction does not apply *after* discretization).

These considerations are concerned with the continuous setting. With a practical implementation in mind, it is necessary to employ a suitable discretization, i.e., one which preserves the salient properties of the Radon transform. We will turn to this matter in the next section.

## Discretization by the Radon approach

### The finite Radon transform

The purpose of this section is to recall the finite Radon transform, originally introduced by Matúš-Flusser [48] and subsequently generalized by different authors [51–53], and to discuss the route towards a computational homogenization scheme [47]. The approach is restricted to cubic voxel grids

3.1 $$\begin{aligned} Y_N = \{0,1,\ldots ,N-1\}^d \end{aligned}$$

in *d* dimensions where we assume that

3.2 $$\begin{aligned} \fbox {the voxel count N is a prime number.} \end{aligned}$$

This restriction is severe when the speed of the fast Fourier transform (FFT) is concerned, as it excludes powers of 2, yet it is indispensable for the development.

We consider the voxel grid (3.1) as a group under addition modulo *N*, i.e., we identify the voxel grid

3.3 $$\begin{aligned} Y_N \equiv (\mathbb {Z}_N)^d \end{aligned}$$

with the *d*-fold Cartesian product of the cyclic group $\mathbb {Z}_N \equiv \mathbb {Z}/ (N\mathbb {Z})$ of order *N*. We are interested in the (non-trivial) cyclic subgroups of the set $Y_N$, which serve as the *discrete equivalent* of the straight lines through the origin, shown in Fig. 1. Recall that a subgroup $C \subseteq Y_N$ is called cyclic provided the entire group *C* can be written in the form

3.4 $$\begin{aligned} C = \{ \varvec{\xi }, 2\,\varvec{\xi }, \ldots , (m-1)\,\varvec{\xi }, \varvec{0}\} \end{aligned}$$

for an element $\varvec{\xi }\in C$, called a generator of the cyclic group, and a positive integer *m*, called the order of the cyclic group. More precisely, the order *m* is the smallest positive integer, s.t. the identity

**Fig. 2:**
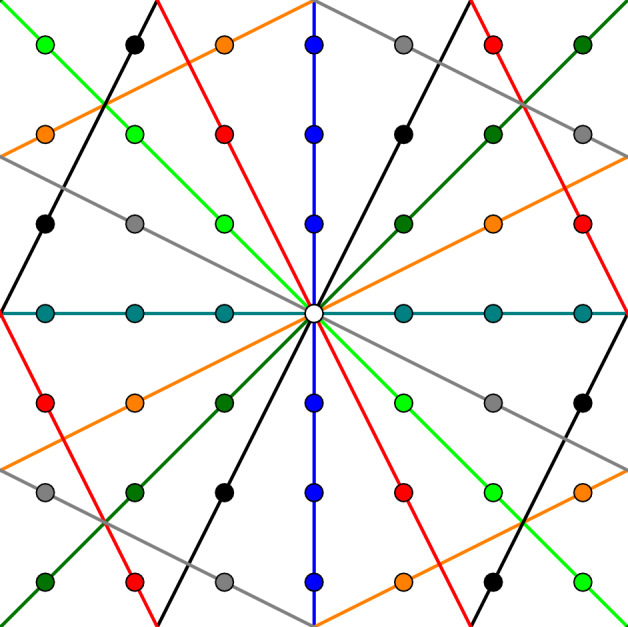
Illustrating the discrete grid $Y_N$ with $N=7$ and two spatial dimensions. The origin, i.e., $\varvec{0}$, is moved to the center (white blob). The eight different cyclic subgroups, which represent discrete lines in this context, are highlighted by different colors

3.5 $$\begin{aligned} m\,\varvec{\xi }= \varvec{0} \quad \texttt {mod} \quad N \end{aligned}$$

holds. To get an intuition of cyclic subgroups, we refer to Fig. 2.

The following essential facts hold for a prime number *N*:

1. Every non-trivial cyclic subgroup has order *N*.
2. Two cyclic subgroups either coincide or intersect precisely at the element $\varvec{0}$.
3. Every non-zero element $\varvec{\xi }\in C\backslash \{\varvec{0}\}$ of a cyclic subgroup is a generator of the subgroup.

These facts are based on elementary number theory (and make use of *N* being prime). For the convenience of the reader, these statements are derived in Appendix B.

With these facts at hand, we can do the following. Every non-zero element $\varvec{\xi }\in Y_N \backslash \{\varvec{0}\}$ gives rise to a cyclic group. By property 1, this group has precisely *N* elements, including $\varvec{0}$. Thus, by property 2, there are

3.6 $$\begin{aligned} \frac{N^d - 1}{N-1} = N^{d-1} + N^{d-1} + \ldots + N + 1 \end{aligned}$$

non-trivial cyclic subgroups in total, see Fig. 2 for a two-dimensional example. In fact, as each of these subgroups has *N* elements, one of which is zero, we have

3.7 $$\begin{aligned} \frac{N^d - 1}{N-1} \cdot (N-1) = N^d - 1 \end{aligned}$$

non-zero elements in the group $Y_N$, as expected. We select a set of generators, see Fig. 2,

3.8 $$\begin{aligned} \mathcal {G}^d_N \subset Y_N \end{aligned}$$

of these groups, i.e., a suitable set $\mathcal {G}^d_N$ of generators consists of precisely $(N^d - 1) / (N-1)$ non-zero elements which give rise to mutually different cyclic subgroups. By property 3, we have a large freedom in selecting the generator set (3.8). In fact, there are $(N-1)^{\frac{N^d - 1}{N-1}}$ possibilities to select a generator set.

For a fixed generator set $\mathcal {G}^d_N$, we may write any *d*-dimensional array, interpreted as a field

3.9 $$\begin{aligned} f: Y_N \rightarrow \mathbb {R}, \quad \varvec{I}\mapsto f[\varvec{I}], \end{aligned}$$

in the form

3.10 $$\begin{aligned} f[\varvec{I}] = f_0 + \sum _{\varvec{\xi } \in \mathcal {G}^d_N} \left( \check{f}_{\varvec{\xi }}[\varvec{I}\cdot \varvec{\xi }] - f_0 \right) , \quad \varvec{I}\in Y_N, \end{aligned}$$

where $f_0$ corresponds to the mean

3.11 $$\begin{aligned} f_0 = \frac{1}{N^d} \sum _{\varvec{I}\in Y_N} f[\varvec{I}] \end{aligned}$$

of the field, and the one-dimensional *N*-periodic arrays are defined

3.12 $$\begin{aligned}  &   \check{f}_{\varvec{\xi }}:\mathbb {Z}_N \rightarrow \mathbb {R}, \quad \check{f}_{\varvec{\xi }}[j] = \frac{1}{N^d} \sum _{k=0}^{N-1} \hat{f}[k \varvec{\xi }] \, e^{2\pi i j k / N}, \nonumber \\  &   \qquad \quad j\in \mathbb {Z}_N, \quad \varvec{\xi }\in \mathcal {G}^d_N, \end{aligned}$$

in terms of the discrete Fourier transform

3.13 $$\begin{aligned} \hat{f}[\varvec{J}] = \sum _{\varvec{I}\in Y_N} f[\varvec{I}] e^{- 2\pi i \, \varvec{I}\cdot \varvec{J}/ N} \end{aligned}$$

of the array *f*. A few remarks are in order.

1. For apparent reasons, the representation (3.10) may be interpreted as a discrete variant of the Radon series (2.17), where $f_0$ corresponds to the spatial average in the continuous case.
2. Instead of the Fourier coefficients (2.11) in the continuous case, the finite Radon series (3.10) involves the discrete Fourier transform (3.13).
3. The derivation of the formula (3.10) is straightforward and follows the continuous case. For the convenience of the reader, the argument is included in Appendix C.
4. There is also a discrete version of the Radon projection (3.12)
  3.14 $$\begin{aligned} \check{f}_{\varvec{\xi }}[j] = \frac{1}{N^{d-1}} \sum _{\varvec{I}\in H^N_{\varvec{\xi },j}} f[\varvec{I}] \end{aligned}$$
  involving the discrete $(d-1)$-dimensional hyperplanes
  3.15 $$\begin{aligned} H^N_{\varvec{\xi },j} = \left\{ \varvec{I}\in Y_N \,\bigg |\, \varvec{I}\cdot \varvec{\xi }= j \,\,  \texttt { mod }\,\, N \right\} , \quad j \in \mathbb {Z}_N, \quad \varvec{\xi }\in \mathcal {G}^d_N. \end{aligned}$$
  The latter formula (and its inverse (3.10)) are called finite Radon transform and were introduced by Matúš and Flusser [48] in a more general setting. Independently, Grigoryan [60–64] used the decomposition (3.10) to speed up the numerical calculation of multi-dimensional discrete Fourier transform, see Grigoryan-Agaian [65]. The argument for establishing the validity of the formula (3.15) parallels the continuous case, see Appendix A. Hence, we omit it.
5. For voxel counts *N* which are no prime numbers, cyclic groups of order less than *N* appear. Moreover, different cyclic groups may intersect in a non-trivial way. We refer to the relevant literature [51–53] for more details. In particular, restricting to prime voxel counts *N* appears imperative to preserve the formal similarity with the Radon series (2.17).

### The finite Radon discretization

Derraz et al. [47] introduced a computational homogenization method based on the finite Radon transform (3.10) for conducting composites. This section comprises the straightforward extension to small-strain mechanics. More precisely, suppose that a stiffness distribution (2.2) is given on the cubic cell

3.16 $$\begin{aligned} Y = [0,L]^d. \end{aligned}$$

Let us fix a prime number *N* of voxels in each direction and consider the discrete stiffness field

3.17 $$\begin{aligned} \mathbb {C}_N: Y_N \rightarrow L(\text {Sym}(d)), \, \mathbb {C}_N[\varvec{I}] = \mathbb {C}(L\varvec{I}/ N) \, \text {for} \, \varvec{I}\in Y_N. \end{aligned}$$

Suppose that a generator set $\mathcal {G}^d_N$ is selected (3.8). Then, for given reference stiffness $\mathbb {C}^0$ and an arbitrary initial field $\varvec{\varepsilon }^0:Y_N \rightarrow \text {Sym}(d)$, the finite Radon version of the basic scheme (2.26)–(2.29) involves the following steps

3.18 $$\begin{aligned} \varvec{\tau }^k[\varvec{I}]&= \left( \mathbb {C}_N[\varvec{I}] - \mathbb {C}^0\right) : \varvec{\varepsilon }^k[\varvec{I}], \quad \varvec{I}\in Y_N, \phantom {\sum _{\varvec{\xi } \in \mathcal {G}^d_N}^{N-1}} \end{aligned}$$

3.19 $$\begin{aligned} \check{\varvec{\tau }}^k_{\varvec{\xi }}[j]&= \sum _{k=0}^{N-1} \hat{\varvec{\tau }}^k[k \varvec{\xi }] \, e^{2\pi i j k / N}, \quad j\in \mathbb {Z}_N, \quad \varvec{\xi }\in \mathcal {G}^d_N, \end{aligned}$$

3.20 $$\begin{aligned} \check{\varvec{\varepsilon }}^{k+1}_{\varvec{\xi }}[j]&= \bar{\varvec{\varepsilon }} + \widehat{\Gamma }^0(\varvec{\xi }) : \check{\varvec{\tau }}^k_{\varvec{\xi }}[j], \phantom {\sum _{\varvec{\xi } \in \mathcal {G}^d_N}^{N-1}} \end{aligned}$$

3.21 $$\begin{aligned} \varvec{\varepsilon }^{k+1}[\varvec{I}]&= \bar{\varvec{\varepsilon }} + \sum _{\varvec{\xi } \in \mathcal {G}^d_N} \left( \check{\varvec{\varepsilon }}^{k+1}_{\varvec{\xi }}[\varvec{I}\cdot \varvec{\xi }] - \bar{\varvec{\varepsilon }} \right) , \quad \varvec{I}\in Y_N. \phantom {\sum _{\varvec{\xi } \in \mathcal {G}^d_N}^{N-1}} \end{aligned}$$

A few remarks are in order.

1. In the three-dimensional setting, $d=3$, Derraz et al. [47] selected the following set of generators:
  3.22 $$\begin{aligned} \mathcal {G}^3_N= &   \{(a,b,1)\,|\, a,b \in {\{0,1,\dots ,N\}}\} \cup \{(a,1,0)\,|\, \nonumber \\  &   a \in {\{0,1,\dots ,N\}}\} \cup \{(1,0,0)\}. \end{aligned}$$
  We will refer to this choice as "the" FRT discretization in the remaining manuscript. The Algorithm (3.18)-(3.21), however, depends on the choice of the generators. In fact, two different non-zero elements $\varvec{\xi }_1$ and $\varvec{\xi }_2$ of the same cyclic group need not give rise to the same Eshelby-Green operators (2.12). For instance, let us consider $N = 3$ and the two vectors
  3.23 $$\begin{aligned} \varvec{\xi }_1 = (1,2,0) \quad \text {and} \quad \varvec{\xi }_2 = (2,1,0). \end{aligned}$$
  We have the identity
  3.24 $$\begin{aligned} \varvec{\xi }_2 = 2\,\varvec{\xi }_1 \,\,  \texttt { mod }\,\, N. \end{aligned}$$
  Thus, both vectors (3.23) are contained in the same cyclic group *C*. The vectors (3.23), however, are not collinear. In particular, the associated Eshelby-Green operators (2.12) do not coincide.
2. To implement the Algorithm (3.18)–(3.21) in practice, it is convenient to avoid the finite-Radon transform (3.10) and just use the fast Fourier transform (FFT) algorithm. Without going into detail, the FRT approach may be integrated into a code running a valid Moulinec-Suquet discretization easily. The only difference lies in the frequencies which are selected for the Eshelby-Green operator (2.12). More precisely, we may decompose the set of non-zero frequencies
  3.25 $$\begin{aligned} Y_N \backslash \{\varvec{0}\} = \bigcup _{C \triangleleft _{\texttt {cyc}} Y_N} C \backslash \{\varvec{0}\} \end{aligned}$$
  as a disjoint union of the cyclic groups *C* avoiding the zero frequency. Then, as we fixed a generator $\varvec{\xi }_C$ for each cyclic subgroup *C*, *each* frequency $\varvec{\eta }\in C \backslash \{\varvec{0}\}$ is treated with the Green’s operator $\Gamma (\varvec{\xi }_C)$. For the Moulinec-Suquet discretization, the $\varvec{\eta }$ frequency is treated with Green’s operator $\Gamma (\varvec{\eta }^{\text {MS}})$ with the vector components ($j=1,2,3$)
  3.26 $$\begin{aligned} \eta ^{\text {MS}}_j = \left\{ \begin{array}{rl} \eta _j, &  j \le N/2,\\ N-\eta _j, & \text {otherwise}. \end{array} \right. \end{aligned}$$
  for odd voxel count *N*.
3. The convergence analysis of the basic scheme [23, 32] remains valid for the finite Radon discretization (3.22) and finite material contrast. Moreover, accelerated solution techniques [20, 25, 27] may be utilized in the usual way with the usual convergence assertions [6] and parameter choices.
4. The finite Radon discretization with the choice of generators (3.22) was demonstrated to have a few salient features [47]. For instance, it was shown that certain laminates can be represented *exactly* by the discretization [47]. In fact, if the laminate normal is contained in the generator set (3.8) and the volume fraction(s) of the laminate are represented exactly by the voxel discretization with voxel count *N*, the finite Radon discretization is able to represent the exact solution for the laminate under consideration. We will also give an example of this phenomenon in Sect. 4.2.
  In contrast, the Moulinec-Suquet discretization [1, 2] is only able to represent a more restricted class of laminates exactly, for instance those where the laminate normal points in the coordinate direction. The reason for this inability is readily read off the Radon series (2.17). To represent a laminate exactly, the lamination normal needs to be collinear with a generator. Moreover, the considered Green’s operator must be homogeneous on the entire generated cyclic group. For the Moulinec-Suquet discretization, this is not the case (see, e.g. Eq. (3.29) below).
5. Taking a close look at the construction of the finite Radon discretization with the generator set (3.22), it appears unreasonable that the discretization scheme is convergent under grid refinement. To be more precise, suppose we wish to represent a general periodic displacement field. Then it is required to provide *all* its Fourier coefficients (2.11). The Moulinec-Suquet discretization considers all frequencies contained in the cube $[-N/2,N/2]^d$. Thus, as $N \rightarrow \infty$, all frequencies will be considered eventually. In contrast, the finite Radon discretization (3.22) considers a number of selected frequencies only, leaving out many required frequencies - for instance those with negative components.
  We will confirm the suspected lack of general convergence with a well-chosen numerical example, see Sect. 4.3.
6. There is a certain connection between the finite Radon approach detailed in this section and a model-order reduction strategy for FFT-based computational micromechanics [66–68], which proposes to operate on a reduced set of Fourier frequencies. In contrast to the Radon approach, which uses the same Eshelby-Green operator for the entire cyclic group, the order-reduction strategy sets certain Eshelby-Green operators to zero. It might be interesting to work out possible synergy effects.

### The consistent Radon discretization

The original discretization [47] based on the finite Radon transform (3.10) turned out to have a few shortcomings. For a start, the discretization turns out to be non-convergent under grid refinement, in general, see Sect. 4.3 for an example. Moreover, the discretization [47] does not reflect certain symmetries present in the microstructure. To be more precise, suppose that the microstructure and the constitutive law satisfy a certain kind of symmetry, i.e., invariance under the action of a symmetry group. Then, it is expected that the effective properties also satisfy the same invariance, i.e., represent the same symmetry. The FRT [47], however, appears to have deficits in this regard, compare Sect. 4.3.

We consider these two deficits to originate from the selection of the generators (3.8) for the cyclic groups which are required to build the finite Radon transform (3.10). To be more precise, for the finite Radon transform (3.10) it is required to select a generator $\varvec{\xi }_C$ for each non-trivial cyclic subgroup *C* of $Y_N$. Any non-zero element of the cyclic subgroup in question can be chosen (by property 3 in Sect. 3.1). Selecting a different generator leads to an *equivalent* Radon transform in the sense that the values of the associated Radon projection (3.12) are permuted.

For the basic scheme (2.15), matters are less simple. In fact, as shown in Eq. (3.26), different generators of the same cyclic group may give rise to *different* Eshelby-Green operators (2.12). Thus, it appears that more care needs to be taken when selecting the appropriate generator of the group. Such a strategy, however, turns out to be insufficient, as well. Let us consider the case $N=5$ and fix the cyclic group *C* generated by the element

3.27 $$\begin{aligned} \varvec{\xi }_C = (1,4,0). \end{aligned}$$

Explicitly, the cyclic subgroup computes as

3.28 $$\begin{aligned} C = \left\{ (1,4,0), (2,3,0), (3,2,0), (4,1,0), (0,0,0) \right\} \end{aligned}$$

In modular arithmetic the following identity holds:

3.29 $$\begin{aligned} (1,4,0) = (1,-1,0) \,\,  \texttt { mod }\,\, 5. \end{aligned}$$

However, none of the elements of the subgroup (3.28) is collinear with the direction $(1,-1,0)$. In particular, this specific direction will never be selected when choosing generators in the way suggested in the previous section.

To sum up, we argue that both the lack of convergence upon grid refinement and the insufficient reflection of underlying symmetry properties is rooted in missing out important Fourier frequencies required for the continuous case (2.11) when constructing the Eshelby-Green frequency $\varvec{\xi }_C^{EG}$ used in the step (2.28) of the basic scheme.

To fix this problem with the Radon discretization, we follow two simple principles:

1. We associate an element $\varvec{\xi }_C$ to each cyclic subgroup $C \triangleleft _{\texttt {cyc}} Y_N$ which generates the cyclic group *C* in modular arithmetic.
2. For each generator $\varvec{\xi }\in \mathcal {G}^d$, the generator set corresponding to the continuous Radon series (2.17), there is an integer $N_0(\varvec{\xi })$, s.t. $\varvec{\xi }\in \mathcal {G}^d_N$ for each $N \ge N_0 (\varvec{\xi })$. Put differently, all generators of the continuous case will be included in the discrete generator set
  3.30 $$\begin{aligned} \mathcal {G}^d_N \subset \mathbb {Z}^d \end{aligned}$$
  eventually, i.e., for a sufficiently high resolution.

The first principle makes sure that certain laminates, i.e., those parallel to the directions $\varvec{\xi }_C$, are represented *exactly* by the Radon discretization, at least provided the volume fractions are resolved exactly. The second principle ensures convergence upon grid refinement. The essential trick is to consider the continuous frequencies, like $\varvec{\xi }= (1,-1,0)$ in Eq. (3.29), which do not naturally appear when considering the set $Y_N$ *without* modular arithmetic.

There is a multitude of ways to satisfy the two stated desired requirements. We propose the following strategy, and call it *consistent* Radon discretization: For a given (prime) voxel count *N*, and a given cyclic group *C*, we select

3.31 $$\begin{aligned} \varvec{\xi }_C = \text {argmin} \left\{ \Vert \varvec{\xi }\Vert _{\infty } \, \bigg | \, \varvec{\xi }\in \mathbb {Z}^d\backslash \{\varvec{0}\}, \quad \text {s.t.} \quad \varvec{\xi }\in C \,\,  \texttt { mod }\,\,N \right\} , \end{aligned}$$

i.e., we choose the representative of all the equivalence classes corresponding to the cyclic group with minimum supremum norm. In case there is more than one minimizer, we select the first one in the lexicographic ordering.

### Implementation

**Algorithm 1:**
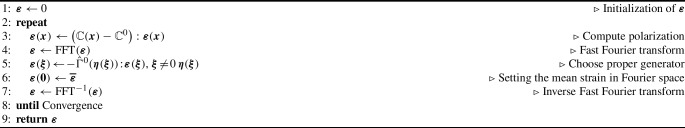
Basic scheme

The goal of this section is to discuss how to implement the finite-radon discretization and its consistent version in the context of FFT-based micromechanics. More precisely, we focus on how to integrate these discretization schemes into an existing FFT-based computational micromechanics code. For this purpose, we discuss the implementation of Moulinec-Suquet’s basic scheme [1, 2] in this framework, as the basic scheme serves as the point of departure for pretty much all the existing extensions.

To minimize the implementation effort, we store the considered frequencies in an array

3.32 $$\begin{aligned} \varvec{\eta }: Y_N \rightarrow \mathbb {Z}^d \end{aligned}$$

which we fill in preprocessing according to the rules (3.22), (3.26) and (3.31). Then, the basic scheme takes the usual form, see Alg. 1.

## Computational investigations

### Setup

**Fig. 3:**
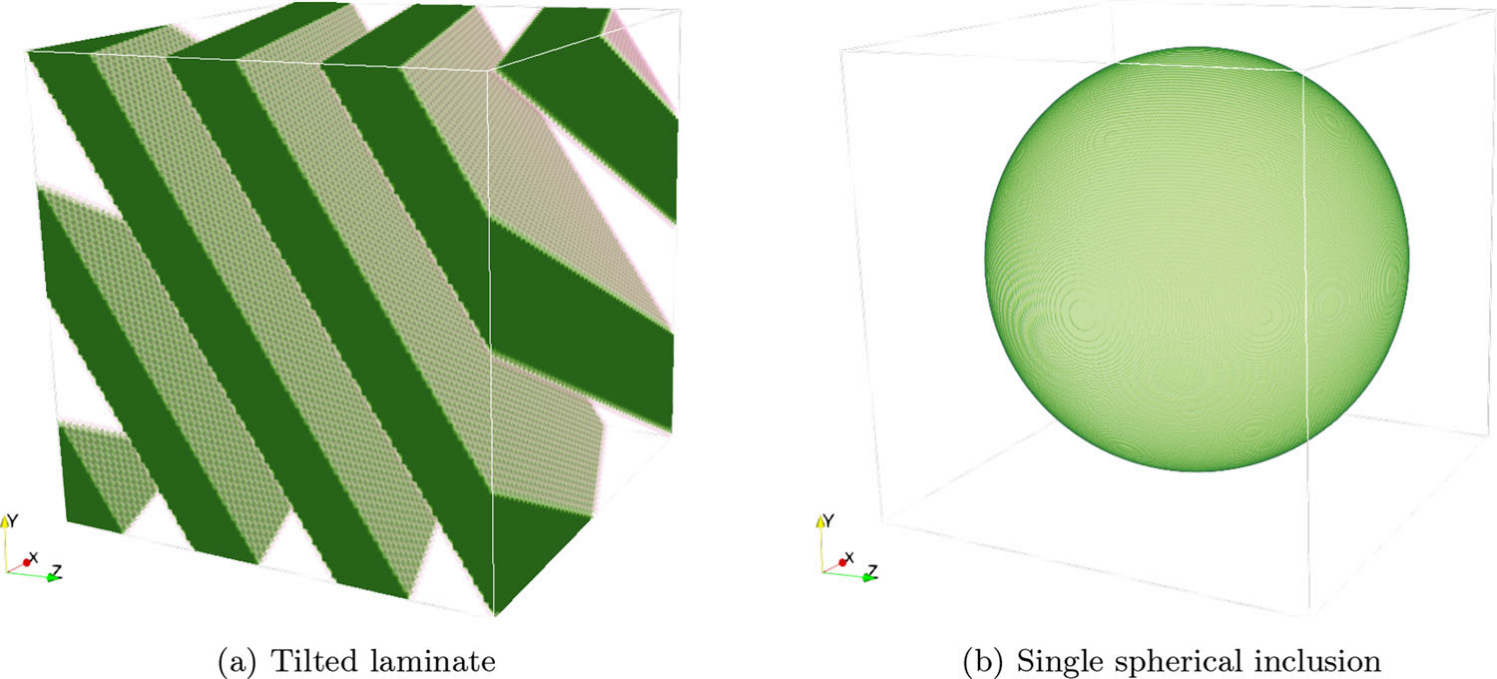
Illustrations of the simple microstructures considered in Sect. 4

The presented discretizations were integrated into an existing FFT-based computational micromechanics code [25] which was written in Python with Cython extensions and parallelized by OpenMP. The computational experiments were run on PC with $2\times 48$-core AMD EPYC CPU with $1024\textrm{GB}$ of RAM.

We employ the conjugate gradient (CG) method [26–28] to solve the ensuing linear systems and use the consistent convergence criterion specified in Schneider et al. [6] to abort the iterations with a tolerance of $10^{-5}$.

The material parameters listed in Table 1 were used for the computational experiments.

**Table 1:** Material parameters considered for the computational experiments

E-glass	$E=72$ GPa	$\nu =0.22$	[69]
Polyamide	$E=2.1$ GPa	$\nu =0.3$	[69]
Quartz sand	$E=66.9$ GPa	$\nu =0.25$	[70, 71]
Waterglass binder	$E=71.7$ GPa	$\nu =0.17$	[71, 72]

### Non-axis aligned laminate microstructures

**Fig. 4:**
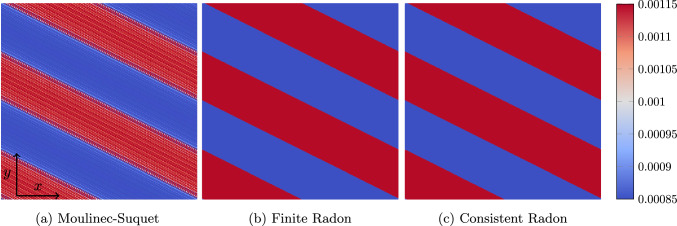
Local strain field ${{\,\mathrm{\varepsilon }\,}}_{xx}$ in a cut-out of a slice at $z=0.5\mu \text {m}$ for the periodic laminate shown in Fig. 3a, a strain loading by $\bar{\varvec{\varepsilon }} = 0.1\%\,\varvec{ e}_x \otimes \varvec{ e}_x$ and $241^3$ voxels

The discretization based on the finite Radon transform (FRT) has the peculiar feature that it permits to represent certain tilted laminates exactly, see Derraz et al. [47]. We investigate this property in the section at hand, and take a deeper look at whether this attractive property does also hold for the consistent Radon discretization (3.31).

For this purpose, we investigate a two-phase laminate with normal $\varvec{ n}=\left( 1,2,3 \right)$ and a volume fraction $\phi = 50\%$, see Fig. 3a. We furnish the phases with the elastic material properties of polyamide and E-glass, see Table 1, and subject the periodic material to a strain loading $\bar{\varvec{\varepsilon }} = 10^{-3}\varvec{ e}_x\otimes \varvec{ e}_x$ for the three discretizations under consideration. The local solution fields are shown in Fig. 4. Both the finite Radon discretization, see Fig. 4b, and the consistent Radon discretization, see Fig. 4c, show a phase-wise homogeneous strain field, as expected for a laminate material. In fact, both discretizations are capable of representing laminates exactly. In contrast, the Moulinec-Suquet discretization, see Fig. 4a, is characterized by ringing artifacts in the direction of the normal orientation.

**Fig. 5:**
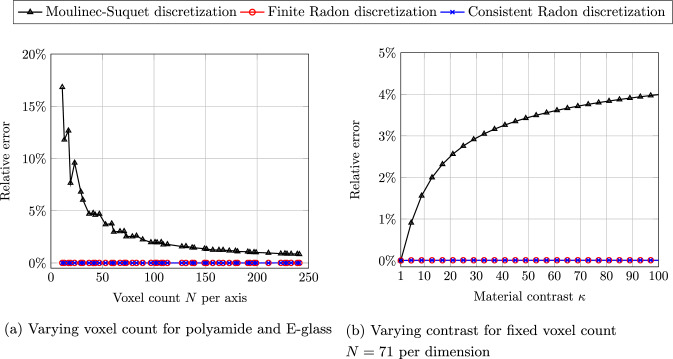
Relative error of the effective stiffness $\mathbb {C}^{\text {eff}}$ for the periodic laminate, see Fig. 3a, and different discretizations

Although the solution fields for both Radon-type discretizations appear to be rather accurate, they actually differ from the analytical solution [73, Ch. 9] – essentially for trivial reasons. The resolution $N=241$ cannot resolve $50\%$ volume fraction exactly. In fact, one phase of the laminate will always comprise 120 voxels, whereas the remaining 121 voxels will always belong to the other phase. Since the discretization error is not of interest here, the analytical solution is computed using the actual volume fractions of the discretizations.

We assess the relative error of the effective stiffness in Fig. 5a, restricting to prime numbers *N* for the voxel count per axis. We observe that the error for both Radon-type discretizations is rather small compared to the Moulinec-Suquet discretization. The latter converges, as expected [74–76]. However, there is a noticeable deviation from the exact solution for low voxel counts.

To take a closer look at the accuracy of the different discretizations, we set up the following scheme. We fix the material parameters of polyamide, i.e.,

4.1 $$\begin{aligned} E_1=E_{\text {PA}} \quad \text {and} \quad \nu _1=\nu _{\text {PA}} \end{aligned}$$

for one phase, and vary Young’s modulus for the second phase

4.2 $$\begin{aligned} E_2=\kappa \, E_1 \quad \text {and} \quad \nu _1=\nu _2, \end{aligned}$$

where $\kappa \ge 1$ quantifies the material contrast.

We fix the voxel count $N = 71$ and vary the material contrast $\kappa$ from 1 to 100. Again, both Radon discretizations have negligible error. In contrast, the Moulinec-Suquet discretization infers an error which increases with the material contrast, yet remains below $4\%$. We also observe that the error of the Moulinec-Suquet discretization appears to follow a $\sqrt{\kappa }$-tendency.

### Single spherical inclusion

**Fig. 6:**
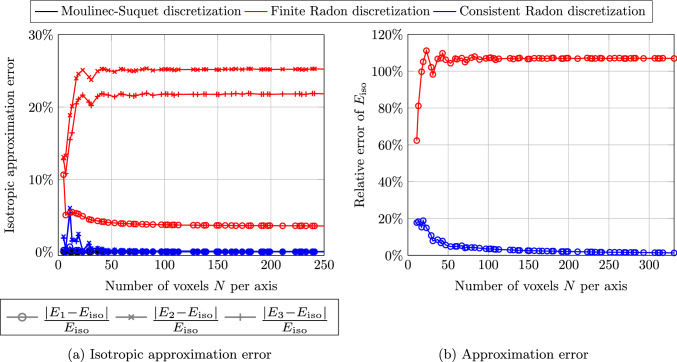
Single sphere inclusion w.r.t. MS-discretization and $401^3$ voxels

After our initial analysis of the finite Radon discretization (3.22), we conjectured that the discretization scheme would not be convergent under grid refinement. However, showing non-convergence can be hard, as other sources of error need to be ruled out. There are preciously few analytic solutions in micromechanics, and a first attempt with Hashin’s coated sphere [77] did not suceed. To be more precise, although the local solution fields did look suspicious, the finite Radon discretization did indeed converge to the exact effective bulk modulus. Therefore, we moved to another example.

The section at hand considers a single spherical inclusion in a cubic volume element, see Fig. 3b. More precisely, a sphere with a diameter of $0.9\textrm{mm}$ is placed at the center of a cubic cell with an edge length of $1\textrm{mm}$. We furnish the matrix with the elastic properties of polyamide, whereas the inclusion is considered as E-glass.

For such a setup, we are not aware of an analytical solution for the effective stiffness. However, we may resort to symmetry arguments to rule out non-physical solutions. To be more precise, due to the setup of the microstructure and the isotropy of the constitutive laws, we expect the effective material behavior to be isotropic. In fact, the microstructure remains unchanged under the reflection symmetries

4.3 $$\begin{aligned} x_j \mapsto L - x_j, \quad j=1,2,3, \end{aligned}$$

where *L* refers to the cell-edge length. Thus, the effective behavior is at least orthotropic. Moreover, the effective behavior is also invariant w.r.t. a permutation of the axes, leading to an eventually isotropic material behavior.

In a first study, we computed the effective stiffness of the single spherical inclusion, see Fig. 3b, and subsequently calculated the orthotropic and the isotropic best-fitting stiffness. Figure 6a details on the deviations of the calculated orthotropic Young’s moduli from the isotropic Young’s modulus. For the discretization by the finite Radon transform, we observe a lack of isotropy of the calculated effective moduli which moreover does not decrease upon (uniform) grid refinement. In fact, the Young’s moduli in the three different spatial directions are predicted to have different values, leading to a systematic error of the FRT discretization. In particular, this example shows that the FRT discretization is *not convergent* under grid refinement. In contrast, the isotropy error for the consistent Radon discretization is much smaller, and decreases upon grid refinement.

The error of the computed isotropic Young’s modulus relative to a high-fidelity computation, i.e., using the Moulinec-Suquet discretization and $401^3$ voxels, is shown in Fig. 6b. We observe that the predicted isotropic Young’s modulus for the finite Radon discretization (3.22) is always at least $60\%$ off. In contrast, we observe a convergence of the effective isotropic Young’s modulus under grid refinement for the consistent Radon discretization (3.31).

**Fig. 7:**
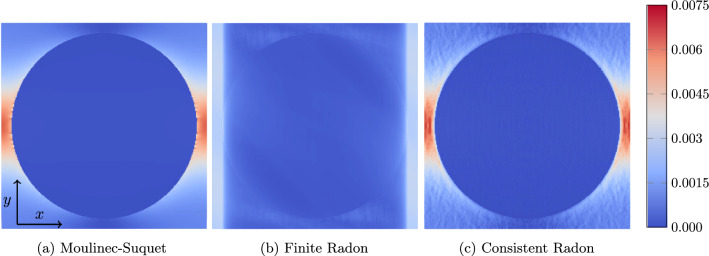
Local strain fields ${{\,\mathrm{\varepsilon }\,}}_{xx}$ at a slice for different discretizations, the single spherical inclusion with $241^3$ voxels and the imposed strain $\bar{\varvec{\varepsilon }} = 0.001\varvec{ e}_x\otimes \varvec{ e}_x$

We close this example by investigating the local solution fields associated to the different discretizations considered. More precisely, we report on the local strain fields in *xx*-direction for a prescribed strain $\bar{\varvec{\varepsilon }} = 0.001\varvec{ e}_x\otimes \varvec{ e}_x$ and $241^3$ voxels, see Fig. 7. The Moulinec-Suquet discretization, shown in Fig. 7a, offers a smooth solution field, where ringing artifacts are visible at the interface of the spherical inclusion and the matrix material. The stiffer spherical inclusion can be clearly distinguished from the more compliant matrix material. For the finite Radon discretization shown in Fig. 7b, the associated strain field appears significantly different. For a start, the edge between the spherical inclusion and the materix is barely recognizable. Moreover, the characteristic regions of high strain at the left and the right center, which we observed for the Moulinec-Suquet discretization, are not present. Instead, there is a region of uniform strain at the left and the right part of the considered slice. For the consistent Radon discretization, shown in Fig. 7c, the strain field is rather similar to the Moulinec-Suquet discretization. However, instead of the ringing artifacts characteristic for the Moulinec-Suquet discretization, we observe what may be described as "noise" in the solution field.

### Bound sand

**Fig. 8:**
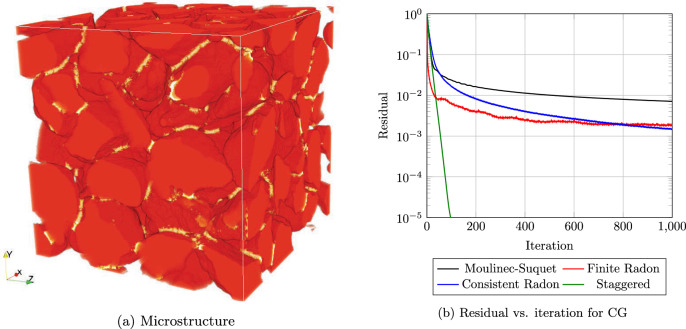
Investigation a sand-binder microstructure [78, 79] with $257^3$ voxels

We investigate the performance of the Radon-type discretizations for microstructures involving pores. Due to the uniform discretizations inherent to FFT-based computational schemes, the classical strategy of eliminating elements representing pores from finite-element simulations is not feasible for such numerical approaches. Instead, these elements need to be accounted for and furnished with a vanishing stiffness. This fact has consequences for computational approaches, as the material contrast experienced by the numerical solution algorithm turns out to be infinite for the scenario at hand.

The FFT-based computational homogenization community spent serious effort to develop improved solution methods to deal with porous materials [32, 80, 81]. In the end, it turned out that the used discretization scheme plays a key role in the performance of the computational approaches. More precisely, the Moulinec-Suquet discretization turned out to lack robustness for porous materials, in general, whereas certain finite-difference [7, 8] and finite-element [10, 11, 82] discretizations lead to a robust convergence, see Schneider [13] for an in-depth discussion. The section at hand investigates the performance of the Radon-type discretizations for such a setup.

We consider microstructures of bound sand, used in foundry applications. More precisely, we operate on a microstructure consisting of $55\%$ quartz sand with $1\%$ waterglass binder. The periodic microstructure, see Fig. 8a, was generated by mechanical contraction [78, 83]. The 64 sand grains are taken from the study of Ettemeyer et al. [79].

For a resolution by $257^3$ voxels, Fig. 8b reports on the residual of the CG method when the material is subjugated to uniaxial extension in *x*-direction. We observe that the two considered Radon-type discretizations fail to get the residual below the $10^{-3}$-mark, showing the characteristic logarithmic "convergence" behavior of the Moulinec-Suquet discretization, which is not suitable for practical applications. As a reference, we included the residual produced by the discretization on a staggered grid [8], which turns out to be robust for such porous materials.

### Fiber reinforced composite

**Fig. 9:**
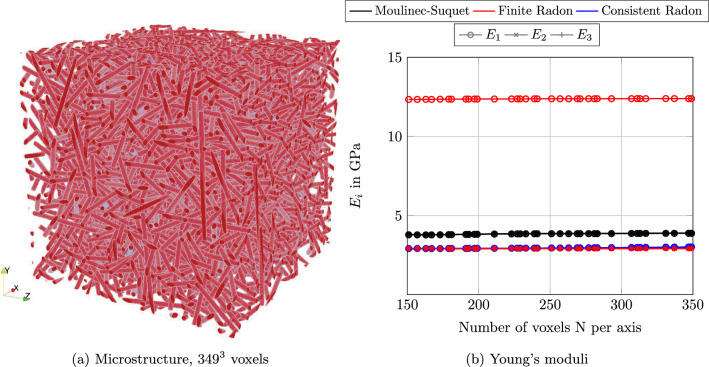
A short glass-fiber reinforced polyamide with isotropic orientation

Last but not least, we consider a microstructure of industrial interest: a polyamide reinforced by short E-glass fibers. We consider fibers with a length $\ell = 250\mu \text {m}$ and a diameter $D = 10\mu \text {m}$ with a fiber-volume content $\phi = 15\%$. The cubic microstructure with an edge length $L=512\mu \text {m}$ and an isotropic orientation, shown in Fig. 9a, was generated by the SAM Algorithm [84]. The individual phases were furnished with the isotropic linear elastic parameters detailed in Table 1.

We used CG to compute the effective stiffness of the composite. We extracted the directional Young’s moduli from the computations. As the fiber orientation was prescribed as isotropic, we expect the effective elastic properties to be isotropic as well. Taking a look at the predicted directional Young’s moduli in Fig. 9b, we observe that the Young’s moduli for the FRT discretization (3.22) do not coincide. Rather, the Young’s modulus $E_1$ in *x*-direction exceeds the other two Young’s moduli $E_2$ and $E_3$ significantly. In contrast, both the Moulinec-Suquet discretization and the consistent Radon discretization predict a roughly isotropic material behavior. The consistent Radon discretization predicts a consistently lower effective Young’s modulus than the Moulinec-Suquet discretization.

**Fig. 10:**
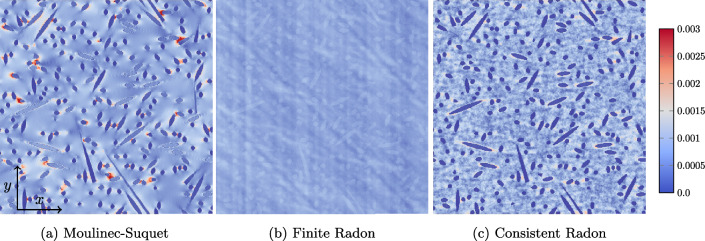
Local strain field ${{\,\mathrm{\varepsilon }\,}}_{xx}$ for the fiber composite shown in Fig. 9a, discretized with $241^3$ voxels and for a strain loading $\bar{\varvec{\varepsilon }} = 0.001 \, \varvec{ e}_x\otimes \varvec{ e}_x$

We investigate the local solution fields in Fig. 10. Similar to the single spherical inclusion, the FRT discretization, shown in Fig. 10b hardly resembles the local strain field. In contrast, the consistent Radon approach, see Fig. 10c, is much more similar to the Moulinec-Suquet discretization, see Fig. 10a, but comes with the characteristic noise.

### Computational effort

As described in Chapter 3.4 the Radon discretizations may be implemented via the FFT. Precomputing the array of frequencies for the Eshelby-Green operator allows us to retain the same algorithmic setup as for the Moulinec-Suquet discretization, see Algorithm 1. Therefore, the computational effort of each iteration coincides with the expense of the Moulinec-Suquet discretization. To avoid external influences, we compare the iterations required for CG to converge for each discretization requires, see Table 2. The microstructures and materials for this analysis are the same as in the previous chapters, and the voxel count of each microstructure is set to $127^3$.

**Table 2:** Comparison of the iteration counts (CG) for different discretizations and microstructures

	Non-axis aligned laminate	Single spherical inclusion	Fiber reinforced composite
Moulinec-Suquet	29	37	41
Finite Radon	3	39	36
Consistent Radon	3	39	29

We observe that the main difference in the computational effort occurs for the laminate microstructure, where both Radon discretizations converge after 3 iterations, whereas the Moulinec-Suquet discretization takes 29 iterations to converge. For the other two microstructures, required iterations are rather close to each other, with a slight exception for the consistent Radon discretization in the case of the fiber reinforced composite.

## Conclusion

The work at hand was directed at understanding the Radon-based approach to computational homogenization introduced by Derraz et al. [47]. We conclude the following items.

1. We provided a stream-lined derivation of the discrete Radon series (2.17) for periodic functions on an arbitrary rectangular cells in any spatial dimension. The argument is rather simple, and may be instrumental in developing AI approaches to computational micromechanics [85, 86]. In fact, a truncation of the Radon series (2.17) may be interpreted as a "shallow" neural network approximation to a periodic field with suitable activation function.
  One interesting aspect of the Radon series (2.17) is that it permits to inter-weave one-dimensional functions into a multi-dimensional function avoiding the traditional tensor-product construction. The Radon series (2.17) gives a convergence guarantee for an arbitrary square-integrable function, yet a large number of terms may be necessary to represent a given function to the desired accuracy. Suitable "deep" neural networks may be used to reduce the necessary number of terms.
2. In an attempt to understand the approach of Derraz et al. [47], we provided a construction of the finite Radon transform (3.12) valid in arbitrary dimensions (but restricted to prime voxel counts and a cubic element). Interestingly, using the detour via the Fourier domain permitted us to re-use the arguments developed for the continuous case in the discrete case with little change.
3. We extended the finite Radon discretization [47] to mechanics and identified its shortcomings. We provided computational examples which demonstrate that the finite Radon discretization may violate expected symmetries and does not converge upon grid refinement, in general. We also introduced an alternative, which we coined *consistent* Radon discretization (3.30) and which is intended to cure some of the deficiencies of the original strategies.
4. To permit developers of FFT-based computational homogenization codes to easily test Radon-type approaches, we provided a simple algorithmic overview of the basic scheme in this framework, see Algorithm 1. With this implementation at hand, the entire bouquet of Lippmann-Schwinger technology becomes available for the Radon-type discretizations, as well.
5. The computational investigations shed some light into why Radon-type approaches may reproduce tilted laminates exactly, but the Moulinec-Suquet discretization does not. Actually, the latter *does* resolve certain laminates, e.g., those whose normal is collinear to the coordinate axes. The principal defect of the Moulinec-Suquet discretization is that the associated Eshelby-Green operator is not homogeneous on the orbit of the laminate normal (in integer coordinates).
6. All in all, the introduced consistent Radon discretization improves upon the finite Radon discretization significantly. For instance, it appears to converge under grid refinement and to respect symmetries of the microstructure. Compared to industrially used discretizations, however, the convergence behavior under grid refinement appears to be slow, exemplified by the distinct difference in the computed effective Young’s moduli for the fiber-reinforced composite. Also, the Radon-type approaches inherit the lack of robustness for porous microstructures characteristic for the Moulinec-Suquet discretization.

## Data Availability

The data that support the findings of this study are available from the corresponding author upon reasonable request.

## Validity of the Radon series

The purpose of this appendix is to show the validity of the Radon-series representation (2.17)

A.1 $$\begin{aligned} f(\varvec{ x}) = \left\langle {f} \right\rangle _Y + \sum _{\varvec{\xi }\in \mathcal {G}^d } \left( \check{f}_{\varvec{\xi }}(\varvec{ x}\cdot \tilde{\varvec{\xi }}) - \left\langle {f} \right\rangle _Y \right) \quad \text {for} \quad \varvec{ x}\in Y, \end{aligned}$$

for a function $f\in L^2(Y)$ with the functions (2.18)

A.2 $$\begin{aligned} \check{f}_{\varvec{\xi }}(s) = \sum _{k \in \mathbb {Z}} \hat{f}(k \, \varvec{\xi }) \, e^{i ks}, \quad s \in [0,2\pi ). \end{aligned}$$

Moreover, we wish to show the alternative form (2.19)

A.3

in case the function *f* is sufficiently smooth, i.e., $f \in W^{1,1}(Y)$, and where we defined (2.20) the sets

A.4 $$\begin{aligned} H_{\varvec{\xi },s} = \left\{ \varvec{ x}\in Y \,\bigg |\, \varvec{ x}\cdot \tilde{\varvec{\xi }} = s \text { mod } 2\pi \right\} \end{aligned}$$

for $$\varvec{\xi }\in \mathcal {G}^d$$ and $$s \in [0,2\pi )$$.

We start by representing the function *f* as a Fourier series (2.10)

A.5 $$\begin{aligned} f(\varvec{ x})= &   \sum _{\xi \in \mathbb {Z}^d} \hat{f}(\varvec{\xi })\,\exp \left( i \, \varvec{ x}\cdot \tilde{\varvec{\xi }}\right) \quad \text {with} \quad \tilde{\varvec{\xi }}_k = \frac{2\pi }{L_k} \xi _k \nonumber \\  &   \quad (k=1,2,\ldots ,d). \end{aligned}$$

We split off the zeroth Fourier coefficient and re-arrange the series

A.6 $$\begin{aligned} \begin{aligned} f(\varvec{ x})&= \hat{f}(\varvec{0}) + \sum _{\xi \in \mathbb {Z}^d \backslash \{\varvec{0}\}} \hat{f}(\varvec{\xi })\,\exp \left( i \, \varvec{ x}\cdot \tilde{\varvec{\xi }}\right) \\&= \hat{f}(\varvec{0}) + \sum _{\varvec{\xi } \in \mathcal {G}^d} \sum _{k \in \mathbb {Z}\backslash \{0\} } \hat{f}(k\,\varvec{\xi })\,\exp \left( i \, k\,\varvec{ x}\cdot \tilde{\varvec{\xi }}\right) . \end{aligned} \end{aligned}$$

As the Fourier coefficient at zero coincides with the average of the function, the definition (A.2) directly implies the formula (A.1).

We turn our attention to the alternative formula (A.3) for the Radon projection. First, notice that it is actually sufficient to show the formula (A.3) for smooth functions *f*, as an approximation argument yields the claim for sufficiently smooth functions. Thus, we restrict to a smooth function $f \in C^\infty _{\text {per}}(Y)$. In this case, its Fourier series (2.10) converges uniformly und absolutely, and we may interchange integration and summation to obtain the equation

A.7

We observe that in case $\varvec{\eta } = k\,\varvec{\xi }$, we obtain

A.8

where we used the definition of the hyperplane (A.4). In case the frequencies $\varvec{\eta }$ and $\varvec{\xi }$ are *not* collinear, it holds

A.9

essentially because a non-constant trigonometric function is integrated over the full period. Returning to the expression (A.7), we deduce

A.10

where we used the definition (A.2).

## Facts about some non-trivial cyclic subgroups

The purpose of this appendix is to establish the following fact for the group $Y_N \equiv (\mathbb {Z}_N)^d$:

1. For a prime number *N* every non-trivial cyclic subgroup has order *N*.
2. Two cyclic subgroups either coincide or intersect precisely at the element $\varvec{0}$.
3. Every non-zero element $\varvec{\xi }\in C\backslash \{\varvec{0}\}$ of a cyclic subgroup is a generator of the subgroup.

These assertions are based on the following fact known from elementary number theory [87]. For integers *a*, *b*, *c* and *n* it holds

B.1 $$\begin{aligned} c\, a = c\, b \,\,  \texttt { mod }\,\,n \quad \text {if and only if} \quad a = b \,\,  \texttt { mod }\,\,n \end{aligned}$$

provided *c* and *n* are coprime. With this assertion (B.1) at hand, we work through the items on our list:

1. Let the subgroup *C* with generator $\varvec{\xi }$ have order *m*. Clearly, the identity
  B.2 $$\begin{aligned} N\, \varvec{\xi }= \varvec{0} \,\,  \texttt { mod }\,\, N \end{aligned}$$
  holds. Thus, the order *m* does not exceed *N*. We argue by contradiction and assume that $m < N$. By definition of the order (3.5) of a cyclic subgroup, the identity
  B.3 $$\begin{aligned} m\,\varvec{\xi }= \varvec{0} \,\,  \texttt { mod }\,\, N \end{aligned}$$
  holds. As the cyclic subgroup is non-trivial, i.e., the inequality $m>0$ holds, there is a non-zero component $\xi _j$ ($j=1,2,\ldots ,d$) of the vector $\varvec{\xi }$. As a special case of Eq. (B.3), the identity
  B.4 $$\begin{aligned} m \, \xi _j = 0 \,\,  \texttt { mod }\,\, N \end{aligned}$$
  holds. As *N* is prime and *m* is less than *N*, *m* and *N* are coprime. Thus, we may infer the result (B.1) for $a = \xi _j$, $b=0$, $c=m$ and $n=N$ to conclude
  B.5 $$\begin{aligned} \xi _j = 0 \,\,  \texttt { mod }\,\, N, \end{aligned}$$
  which is a contradiction to the assumed inequality $m < N$.
2. This property follows directly from property 3, as each non-zero element generates the whole cyclic subgroup.
3. Each non-zero element generates a non-trivial cyclic subgroup of the considered cyclic group. By property 1., the order of the generated subgroup is *N*. Thus, the entire group is generated.

## On the finite Radon transform

In this appendix, we wish to derive the formula

C.1 $$\begin{aligned} f[\varvec{I}] = f_0 + \sum _{\varvec{\xi } \in \mathcal {G}^d_N} \left( \check{f}_{\varvec{\xi }}[\varvec{I}\cdot \varvec{\xi }] - f_0 \right) , \quad \varvec{I}\in Y_N, \end{aligned}$$

for the finite Radon transform (3.10) with the mean (3.11)

C.2 $$\begin{aligned} f_0 = \frac{1}{N^d} \sum _{\varvec{I}\in Y_N} f[\varvec{I}] \end{aligned}$$

and the arrays (3.12)

C.3 $$\begin{aligned}  &   \check{f}_{\varvec{\xi }}:\mathbb {Z}_N \rightarrow \mathbb {R}, \quad \check{f}_{\varvec{\xi }}[j] = \frac{1}{N^d} \sum _{k=0}^{N-1} \hat{f}[k \varvec{\xi }] \, e^{2\pi i j k / N}, \nonumber \\  &   \qquad \quad j\in \mathbb {Z}_N, \quad \varvec{\xi }\in \mathcal {G}^d_N. \end{aligned}$$

The line of argument closely follows the continuous case, see Appendix A. We start from the following formula for the inverse discrete Fourier transform

C.4 $$\begin{aligned} f[\varvec{I}] = \frac{1}{N^d} \sum _{\varvec{J}\in Y_N} \hat{f}[\varvec{J}] \, e^{2\pi i \, \varvec{I}\cdot \varvec{J}/ N}. \end{aligned}$$

Separating the zeroth frequency and splitting the non-zero frequencies into the (essentially disjoint) cyclic subgroups, we obtain the formula

C.5 $$\begin{aligned} \begin{aligned} f[\varvec{I}]&= \frac{1}{N^d} \sum _{\varvec{J}\in Y_N} \hat{f}[\varvec{J}] \, e^{2\pi i \,\varvec{I}\cdot \varvec{J}/ N}\\&= \frac{\hat{f}[\varvec{0}]}{N^d} + \frac{1}{N^d} \sum _{\varvec{J}\in Y_N \backslash \{\varvec{0}\}} \hat{f}[\varvec{J}] \, e^{2\pi i \, \varvec{I}\cdot \varvec{J}/ N}\\&= \frac{\hat{f}[\varvec{0}]}{N^d} + \frac{1}{N^d} \sum _{C \triangleleft _{\text {cyc}} Y_N} \sum _{\varvec{J}\in C \backslash \{\varvec{0}\}} \hat{f}[\varvec{J}] \, e^{2\pi i \, \varvec{I}\cdot \varvec{J}/ N}\\&= \frac{\hat{f}[\varvec{0}]}{N^d} + \frac{1}{N^d} \sum _{\varvec{\xi } \in \mathcal {G}^d_N} \sum _{k = 1}^{N-1} \hat{f}[k\,\varvec{\xi }] \, e^{2\pi i \, k\varvec{I}\cdot \varvec{\xi } / N}\\&= \frac{\hat{f}[\varvec{0}]}{N^d} + \sum _{\varvec{\xi } \in \mathcal {G}^d_N} \frac{1}{N^d} \left[ \sum _{k = 0}^{N-1} \hat{f}[k\,\varvec{\xi }] \, e^{2\pi i \, k\varvec{I}\cdot \varvec{\xi } / N} - \hat{f}[\varvec{0}]\right] \\&= \frac{\hat{f}[\varvec{0}]}{N^d} + \sum _{\varvec{\xi } \in \mathcal {G}^d_N} \left[ \frac{1}{N^d} \sum _{k = 0}^{N-1} \hat{f}[k\,\varvec{\xi }] \, e^{2\pi i \, k\varvec{I}\cdot \varvec{\xi } / N} -\frac{\hat{f}[\varvec{0}]}{N^d} \right] . \end{aligned} \end{aligned}$$

We notice from the definition (3.13) of the discrete Fourier coefficients

C.6 $$\begin{aligned} \hat{f}[\varvec{0}] = \sum _{\varvec{I}\in Y_N} f[\varvec{I}] e^{- 2\pi i \, \varvec{I}\cdot \varvec{0} / N} = \sum _{\varvec{I}\in Y_N} f[\varvec{I}] = N^d \, f_0, \end{aligned}$$

where we used equation (C.2). Together with the definition of the Radon projections (C.3), we thus obtain

C.7 $$\begin{aligned} \begin{aligned} f[\varvec{I}]&= \frac{\hat{f}[\varvec{0}]}{N^d} + \sum _{\varvec{\xi } \in \mathcal {G}^d_N} \left[ \frac{1}{N^d} \sum _{k = 0}^{N-1} \hat{f}[k\,\varvec{\xi }] \, e^{2\pi i \, k\varvec{I}\cdot \varvec{\xi } / N} -\frac{\hat{f}[\varvec{0}]}{N^d} \right] \\&= f_0 + \sum _{\varvec{\xi } \in \mathcal {G}^d_N} \left( \check{f}_{\varvec{\xi }}[\varvec{I}\cdot \varvec{\xi }] - f_0 \right) , \end{aligned} \end{aligned}$$

which is what we wanted to show.
